# Journeys of a Lifetime: Applying Antiracist Pedagogy to Health Disparities Across the Life Course

**DOI:** 10.1093/geroni/igaf122.689

**Published:** 2025-12-31

**Authors:** Kyoko Kishimoto, Rona Karasik

**Affiliations:** St. Cloud State University, St. Cloud, Minnesota, United States; Saint Cloud State University, Saint Cloud, Minnesota, United States

## Abstract

Pervasive health disparities among ethnic and racial groups throughout the life-course and across generations are well documented and become amplified as they intersect with factors of age and gender (Martinez, 2024; Steward et al., 2025). Despite this knowledge, racial disparities in all aspects of health continue to persist, suggesting simple acknowledgement is insufficient to address them. Rather, educators must guide the next generation of researchers and practitioners to uncover root causes of these disparities – an essential step toward effecting social change. A significant barrier to this process is a lack of historical knowledge and policy context among students and many educators (Karasik & Kishimoto, 2018; 2022). This challenge is exacerbated by current political attacks on DEI, which by design, create confusion and fear to even talk about health disparities – much less try to alleviate them. Silencing the problem, however, does not make it go away. Therefore, new and enhanced strategies are needed to engage and support students in exploring the sources and impact of these disparities. Using an antiracist pedagogical approach, this project pairs an extensive annotated timeline of key historical events and multi-media resources with health care related policies/programs in a digitized format – allowing students to follow historically accurate, fictionalized life-story vignettes to their current circumstances, while also providing opportunities to extrapolate potential future outcomes. In addition to providing an analysis of the underlying principles and inner workings of this interactive interface, this presentation offers insight into the student learning experience and strategies for next steps.

